# Fatigue and sleep quality improvement through complete decongestive therapy in postmastectomy lymphedema: An investigative analysis

**DOI:** 10.1007/s00520-024-08590-4

**Published:** 2024-05-29

**Authors:** Songül Keskin Kavak, Engin Eren Kavak

**Affiliations:** 1 Department of Physical Therapy And Rehabilitation, Ankara Dr. Abdurrahman Yurtaslan Training And Research Hospital, Ankara, Turkey; 2 Department of Medical Oncology, Ankara University Faculty of Medicine, Ankara, Turkey

**Keywords:** Complete decongestive therapy, The LYMQOL-Arm Scale, Hirai Cancer Fatigue Scale, Pittsburgh Sleep Quality Index, Postmastectomy lymphodema syndrome

## Abstract

**Objective:**

To evaluate the effects of complete decongestive therapy (CDT) on cancer-related fatigue, sleep quality, and lymphedema-specific quality of life using validated and reliable questionnaires in cancer patients being commendable.

**Material and methods:**

This prospective study includes 94 patients who had postmastectomy lymphedema syndrome. The demographic characteristics of the patients were recorded. The participants’ stages of lymphedema (The International Society of Lymphology), Hirai Cancer Fatigue Scale (HCFS) score, Pittsburgh Sleep Quality Index (PSQI) Global score, lymphedema-specific quality of life questionnaire (LYMQOL-ARM) score, and Global health status were recorded before and after CDT.

**Results:**

The mean age of the patients was 58.49 ± 10.96 years. Strong correlations were found between the severity of edema and global health status. There was a significant positive relationship between the HCFS score, PSQI Global score, LYMQOL-ARM score, and CDT. After decongestive physiotherapy, the majority of the lymphedema stages were downstaging (*p* < 0.05), respectively. There was also a trend toward improvement in general well-being (*p* < 0.05).

**Conclusion:**

Cancer-related fatigue and sleep disturbance can persist for years after surgery in women with breast cancer. This can negatively affect the patient physically, socially and cognitively. Our study, which is the first study to investigate the HCFS score in postmastectomy patients and the relationship between PSQI Global score and CDT. The findings identify the risk factors that affect these outcomes in women with lymphedema and can provide valuable insights for targeted interventions and improved patient care.

## Introduction

Breast cancer is the most common cancer in women worldwide. According to 2020 GLOBOCAN data, it accounts for 11.7% of all cancer cases in women, making it the most common cancer [1]. This statistic highlights the fact that breast cancer is a significant health problem among women and is widespread throughout the world.

Postmastectomy lymphedema syndrome is a chronic and progressive complication that occurs in patients who have undergone lymph node dissection, surgery or radiotherapy for breast cancer. It involves the accumulation of protein-rich fluid in the interstitial spaces and is caused by the disruption of lymphatic drainage [2]. If left untreated, lymphedema can lead to chronic inflammation, cellulite, pain, fatigue, loss of productivity, cosmetic disfigurement and functional impairment of the affected limb, significantly affecting daily life [3]. Incidence rates for postmastectomy lymphoedema can vary widely, but it is estimated that around 30% of women diagnosed with breast cancer will develop postmastectomy lymphoedema [4]. This condition highlights the importance of understanding and managing the potential complications associated with breast cancer and its treatment, including the risk of lymphoedema. Early detection and appropriate management strategies are critical to improving the quality of life for people with postmastectomy lymphoedema.

Currently, the gold standard approach for lymphedema treatment is Complete Decongestive Therapy (CDT). CDT is a comprehensive treatment method that includes skin care, manual massage techniques, compression therapy (double-layer bandaging), and lymphedema exercises [5].

Cancer-related fatigue is one of the symptoms that can persist for years in women with breast cancer, even after the completion of adjuvant therapy. The etiology of cancer-related fatigue can be attributed to physiological, psychological and anatomical factors, and it can have a significant impact on a patient’s health-related quality of life both before and after surgery [6]. Symptoms of cancer-related fatigue include low energy, myalgia (muscle pain), arthralgia (joint pain), cancer cachexia (severe weight loss and muscle wasting), immobility, difficulty with attention and concentration, inability to perform daily tasks, persistent fatigue whether active or at rest, excessive or insufficient sleep, and spending more than 24 h in bed [7]. These symptoms can have a significant impact on a patient’s physical and emotional well-being and may require appropriate management and support from healthcare professionals. This study examined the impact of pessimism, depression, fatigue and pain on functional health-related quality of life in breast cancer patients. Fatigue was found to negatively affect physical, social, cognitive and role aspects of health-related quality of life, suggesting a significant association between fatigue and health-related quality of life [8]. This highlights the importance of addressing and managing fatigue in breast cancer patients to improve their overall well-being and health-related quality of life during and after treatment.

One of the most common symptoms experienced by cancer patients is sleep disturbance. Studies have shown that insomnia can affect all dimensions of health-related quality of life in patients undergoing cancer treatment, both before and after diagnosis [9, 10]. A study by Tamam et al. looked at 163 women with postmastectomy lymphedema syndrome. The study found a significant correlation between patients’ health-related quality of life and sleep quality, and both were statistically reduced after breast surgery [11]. According to the available literature, there is little comprehensive research on the effect of CDT on sleep by increasing lymphatic circulation, with only one case study found by [12].

The primary aim of the study is to evaluate the effects of CDT on post-mastectomy lymphedema-related fatigue, sleep quality and quality of life. The secondary aim of the study is to improve patients’ self-care and quality of life by contributing to the management of complications that may develop after cancer treatment. The strength of the study is that, according to the available literature, this is the first time that the Hirai Cancer Fatigue Scale has been evaluated in post-mastectomy lymphedema patients.

## Material and methods

This prospective study was conducted between January 2023 and June 2023 at a tertiary healthcare institution/oncology training and research hospital. The study included patients diagnosed with local/locally advanced breast cancer who underwent modified radical mastectomy and developed posttreatment lymphedema (painless swelling, edema, and tissue changes in the affected limb) in the upper extremity. These patients had completed their adjuvant treatment (chemotherapy and/or radiotherapy, hormone therapy) and/or were continuing hormone therapy before enrolment. Exclusion criteria; multiple metastases, advanced heart failure, arteriovenous circulation disorder, skin lesions such as cellulitis and lymphangitis. Patients’ demographic and clinical information, including age, education level, marital status, socioeconomic status, social support, living arrangements, BMI (body mass index), smoking status, alcohol consumption, duration of lymphoedema, and use of hormone therapy, was recorded to assess their characteristics.

Each patient received a standardised treatment protocol that included skin care, manual massage techniques, compression therapy (multi-layer bandaging) and lymphedema exercises, forming a multi-component treatment method known as the complete decongestive therapy (CDT) programme [5]. All patients underwent an initial assessment by a clinician specialising in lymphedema. After 3 weeks and 21 sessions of exercise and instruction, a final assessment was carried out and the relevant assessment scales were completed by the treating clinician. Treatment was planned for 21 sessions over 3 weeks, 7 days a week. Patients were assessed with questionnaires one day before and one day after treatment. The effects of CDT on lymphedema stage, patients’ health-related quality of life, fatigue and sleep quality were assessed in the trial.

### Sample size

Due to the lack of data availability, sample size was estimated using traditional power analysis with a descriptive design (α = 0.5, effect size = 0.4 and confidence interval = 80%). The required minimum sample size was 90 participants. Therefore, 94 women were included in the study to compensate for the withdrawal rate.

### Patient evaluation methods

- The International Society of Lymphology(ISL)

The International Society of Lymphology [13] scale is utilized for staging lymphedema, and each patient is assessed based on the following stages [14]:

- **Stage 0:** This is a subclinical condition in which there is no apparent swelling despite impairment of lymphatic transport.
- **Stage 1:** This is an early stage of the disease with visible accumulation of protein-rich tissue fluid. The swelling may exhibit pitting, i.e. pressing with the thumb causes an indentation that persists for some time. The swelling subsides with elevation of the affected limb.
- **Stage 2:** This stage is characterised by an increase in swelling that does not subside with elevation. Initially, pitting is evident, but over time the tissue continues to proliferate and harden, making pitting more difficult to induce.
- **Stage 3:** At this stage, the tissue becomes harder (more fibrotic) and pitting is absent. There is a potential for further enlargement of the lymphedema, sometimes to extreme proportions. In additional, there are skin changes such as thickening, hyperpigmentation, increased (deepened) skin folds, fat deposits and warty proliferations.

b) The Lymphedema Quality of Life Questionnaire-Arm Scale (The LYMQOL-Arm Scale)

The LYMQOL-Arm scale, a lymphedema-specific quality of life questionnaire, was used to assess health-related quality of life in patients with lymphedema. The LYMQOL-Arm was developed by Keeley et al. [15]. To evaluate the impact of lymphedema on health-related quality of life and is a valid and reliable questionnaire. It consists of 21 questions divided into five sections: arm function, appearance, symptoms, emotional well-being, and the last section assesses overall health-related quality of life. Each item in the first 20 questions is scored on a scale of 1 to 4 (1 = Not at all, 2 = A little, 3 = Quite a bit, 4 = A lot). In the last section, which evaluates the general health-related quality of life (**Global Health Status**), the patient is asked to give a value between 0 and 10. When the patient marks 0 (zero) points, it indicates a very poor quality of life, while a score of 10 indicates an excellent quality of life. The total score of the questionnaire was calculated separately for the first 20 questions and the 21st question. A high score for the first 20 questions and a low score for the 21st question indicate a poor health-related quality of life.

c) Hirai *Cancer* Fatigue Scale (HCFS)

The ‘Hirai Cancer Fatigue Scale’, developed by Hirai and colleagues in 2015, was used to assess cancer-related fatigue in our study. This scale is designed to measure the fatigue experienced by cancer patients and is based on a 5-point Likert scale. Each item on the scale is rated from 1: “Not at all,” 2: “Slightly,” 3: “Somewhat,” 4: “Very,” to 5: “Extremely.” The scale consists of three subscales: the physical-mental subscale, the activity-related sensitivity subscale, and the cognitive sensitivity subscale. Each subscale assesses different aspects of cancer-related fatigue. There is no specific cut-off point for the scale. The minimum score on the scale is 15, indicating the lowest level of fatigue, while the maximum score is 75, indicating the highest level of fatigue. Higher scores on the scale represent increased fatigue, while lower scores indicate lower levels of fatigue experienced by cancer patients [16].

d) Pittsburgh Sleep Quality Index (PSQI)

In our study, we assessed patients’ sleep quality using the Pittsburgh Sleep Quality Index (PSQI). The PSQI is a validated and reliable scale for assessing sleep quality in women with breast cancer. The scale consists of 24 questions, 18 of which are scored. For this study, sleep quality was categorised into three groups: low, moderate and high sleep quality. The PSQI consists of seven components that provide a comprehensive assessment of sleep quality. These components are: sleep quality, sleep latency (the time it takes to fall asleep), sleep duration, habitual sleep efficiency, sleep disturbances, use of sleep medication and daytime dysfunction [17].

### Statistical analysis

The data of the study were recorded with the Statistical Package for the Social Sciences (SPSS) version 25 (IBM corporation, New York, USA) program and statistical analyses were made. Frequency tables and descriptive statistics were used to interpret the findings for statistical analysis. Categorical variables were analyzed using the Kruskal–Wallis test. The “Student’s *t*-test” *t*-table value was used to compare the scale score averages of the independent variables with normal distribution. Pearson correlation “*r*” coefficient was used for normally distributed averages in the comparison of the relationship between the scale point averages according to the research question. *P* values < 0.05 were considered statistically significant.

### Ethics

Individuals who met the eligibility criteria and applied to the outpatient clinics of the Oncological Physical Medicine and Rehabilitation Department of Dr. Abdurrahman Yurtaslan Training and Research Hospital were evaluated for participation in the study. Those who met the inclusion and exclusion criteria expressed their willingness to participate in the study and gave written informed consent in accordance with the ethical principles outlined in the Declaration of Helsinki.

Ethical approval for this study was obtained from the Oncology Training and Research Hospital Clinical Research Ethics Committee (decision no: 06, date: 12.01.2023). Written informed consent was obtained from all patients after providing them with detailed information about the study.

## Results

**Fig. 1:**
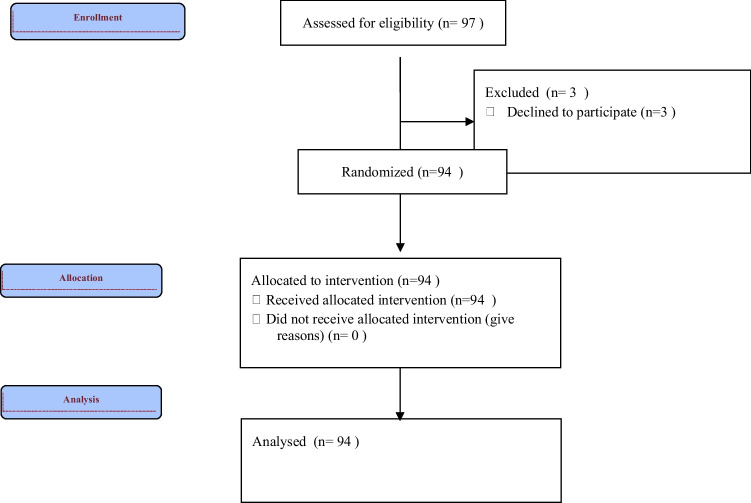
Flow chart of the study

Flow chart of the study was given in Fig. 1.

A total of 94 patients diagnosed with breast cancer were included in the study, with a mean age of 58.49 ± 10.96 years, a mean body mass index (BMI) of 29.96 ± 5.57, 81 (86.2%) of the patients had a BMI greater than 25 kg/m^2^ and a mean post-mastectomy duration of 38.40(1–200) months. Regarding their level of education, 25(26.6%) had no formal education, 33(35.1%) had primary education, 21(22.3%) had secondary education, and 15(16%) had tertiary or other education. In addition, 67(71.3%) were married and 27(28.7%) were unmarried. Regarding the 3 different stages of the disease, 5(5.3%) of the women were in stage I, 45(47.9%) in stage II and 44(46.8%) in stage III. Table 1 summarises the demographic and clinical characteristics of all patients.

**Table 1:** Demographics and clinical features of the study participants

	*N* = 94
Age (years) mean ± SD	58.49 ± 10.96
BMI (kg/m^2^) mean ± SD	29.96 ± 5.57
BMI, *n* (%)	
≥ 25	81(86.2)
< 25	13(13.8)
Married, *n* (%)	67(71.3)
With whom she lived, *n* (%)	
Alone	14(14.9)
Husband	18(19.1)
Children	13(13.8)
Husband&Children	48(51.1)
Parents	1(1.1)
Level of Education, *n* (%)	
İlliterate	14(14.9)
Semiliterate	11(11.7)
Primary school	33(35.1)
High school	21(22.3)
University	15(16)
Economic status, *n* (%)	
High	5(5.3)
Medium	30(31.9)
Low	59(62.8)
Smoker, n (%)	13(13.8)
Hormone theraphy, *n* (%)	
None	21(22.3)
Tamoxifen	28(29.8)
Letrozole	37(39.4)
Anastrazole	8(8.5)
Duration of Lymphedema (months) mean(min–max)	38.40(1–200)
Stages of lymphedema, *n* (%)	
Stage I	5(5.3)
Stage II	45(47.9)
Stage III	44(46.8)
HCFS score, mean ± SD	47.64 ± 15.54
PSQI Global Score, mean ± SD	8.21 ± 4.28
LYMQOL- ARM score, mean ± SD	59.10 ± 17.71
Global health status, mean ± SD (LYMQOL-ARM scale)	5.13 ± 1.83

*HCFS*, Hirai Cancer Fatigue Scale; *PSQI*, Pittsburgh Sleep Quality Index; *LYMQOL*, Lymphedema-specific Quality of Life

Table 2 outlines the factors linked to Global Health Status and PSQI Global Scores. The results of the multivariate regression analysis indicate that marital status and family status (especially with children) emerged as significant factors in predicting global health (overall health) status among patients (*p* < 0.05). However, age, BMI, educational level, economic status, and the stages of lymphedema in patients did not significantly predict global health (overall health) status and PSQI Global Scores. These findings provide insights into the potential influence of marital status on the overall health status of the patients, while other factors in the analysis did not show a significant predictive relationship with global health and sleep quality.

**Table 2:** Predictors of Global Health Status and PSQI Global Score of breast cancer patients

	Global Health Status		PSQI Global Score	
	B (97.5% CI)	*p*-value	B (97.5% CI)	*p*-value
Age	0.01(− 0.03 to 0.05)	0.62	0.03(− 0.06 to 0.14)	0.45
BMI	− 0.02(− 0.09 to 0.05)	0.57	0.02(− 0.16 to 0.21)	0.78
Marital status	− 1.13(− 2.08 to − 0.18)	**0.02**	0.27(− 2.01 to 2.56)	0.81
Family status				
Husband	− 0.23(− 1.45 to 0.99)	0.70	− 0.97(− 4.00 to 2.05)	0.52
Children	− 1.6(− 2.98 to − 0.35)	**0.01**	0.16(− 3.11 to 3.44)	0.92
Husband and children	0.29(− 0.74 to 1.33)	0.57	− 1.58(− 4.16 to 1.00)	0.22
Parents	− 3.28(− 6.83 to 0.26)	0.06	3.85(− 4.95 to 12.66)	0.53
Educational status	− 0.75(− 2.19 to 0.68)	0.29	1.56(− 1.99 to 5.11)	0.38
Economic status	− 0.15(− 2.05 to 1.75)	0.49	− 1.49(− 6.29 to 3.29)	0.53
Stages of lymphedema	− 0.75(− 2.19 to 0.68)	0.21	0.25(− 1.59 to 2.10)	0.78

* Multivariate regression analysis, *PSQI*, Pittsburgh Sleep Quality Index

As shown in Table 3, the analysis of HCFS score, PSQI Global score, LYMQOL-ARM score and Global health status according to the stage of lymphedema showed that the stage I lymphedema patients (*n* = 5) reported the best values HCFS score, PSQI Global score, LYMQOL-ARM score, Global health status (50.40 ± 14.77, 9.60 ± 4.21, 53.80 ± 7.82, and 6.20 ± 0.83, respectively). The stage II lymphedema patients (*n* = 45); HCFS score, PSQI Global score, LYMQOL-ARM score, Global health status (43.31 ± 15.60, 7.38 ± 4.14, 57.47 ± 18.16 and 5.51 ± 2.00, respectively); HCFS score, PSQI Global score, LYMQOL-ARM score, Global health status (51.75 ± 14.67, 8.91 ± 4.35, 61.36 ± 17.99, and 4.61 ± 1.60, respectively).

**Table 3:** HCFS score, PSQI Global score, LYMQOL- ARM score, Global health status According to the Stages of Lymphedema before CDT

	Stage I(*n* = 5)	Stage II(*n* = 45)	Stage III(*n* = 44)	p*value
HCFS score, mean ± SD	37.00 ± 14.47	32.26 ± 12.55	36.22 ± 10.93	0.262
PSQI Global Score, mean ± SD	9.60 ± 4.21	7.38 ± 4.14	8.91 ± 4.35	0.183
LYMQOL-ARM score, mean ± SD	53.80 ± 7.82	57.47 ± 18.16	61.36 ± 17.99	0.465
Global health status, mean ± SD	6.20 ± 0.83	5.51 ± 2.00	4.61 ± 1.60	**0.027**

* ANOVA, 1 and 2 way. *HCFS*, Hirai Cancer Fatigue Scale; *PSQI*, Pittsburgh Sleep Quality Index; *LYMQOL*, Lymphedema-specific Quality of Life; *CDT*, Complete Decongestive Therapy

Upon analyzing the differences between the 3 stages of lymphedema with the help of the Kruskal–Wallis test in the health-related quality of life; these findings pointed to certain noteworthy statistical differences between the 3 stages of lymphedema (*p* < 0.05) stage III lymphedema patients showed the lowest health-related quality of life values when compared to those of stage I and stage II lymphedema patients As for LYMQOL-ARM and HCFS scores, the stage III lymphedema patients showed worse values than the stage I and stage II lymphedema patients.

Changes in HCFS score, PSQI Global score, LYMQOL-ARM score, and Global health status from baseline to the end of CDT treatment are shown in Table 4. All groups’ results showed a significant improvement (*p* < 0.05).

**Table 4:** Changes in HCFS score, PSQI Global score, LYMQOL-ARM score, Global health status from baseline to the end of CDT treatment

	Baseline Mean ± SD	After CDT treatment Mean ± SD	Mean difference (95% CI)	p* value
HCFS score	47.64 ± 15.54	34.37 ± 11.96	11.35 to 15.18	< 0.001
PSQI Global Score	8.21 ± 4.28	5.70 ± 2.35	2.01 to 3.00	< 0.001
LYMQOL-ARM score	59.10 ± 17.71	43.05 ± 11.88	13.89 to 18.18	< 0.001
Global health status	5.13 ± 1.83	6.95 ± 1.32	− 2.15 to − 1.47	< 0.001

* Paired samples *t*-test, *HCFS*, Hirai Cancer Fatigue Scale; *PSQI*, Pittsburgh Sleep Quality Index; *LYMQOL*, Lymphedema-specific Quality of Life; *CDT*, complete decongestive therapy

After evaluating the patients based on their lymphedema stage following CDT, the results showed the following changes; among the 5 patients who were initially in stage 1 before treatment, all of them remained in stage 1 after CDT. Out of the 45 patients who were initially in stage 2, 35 of them improved to stage 1, while 10 remained in stage 2. Among the 44 patients who were initially in stage 3, 8 of them improved to stage 1, 34 improved to stage 2, and 2 patients remained in stage 3.

**Chart 1:**
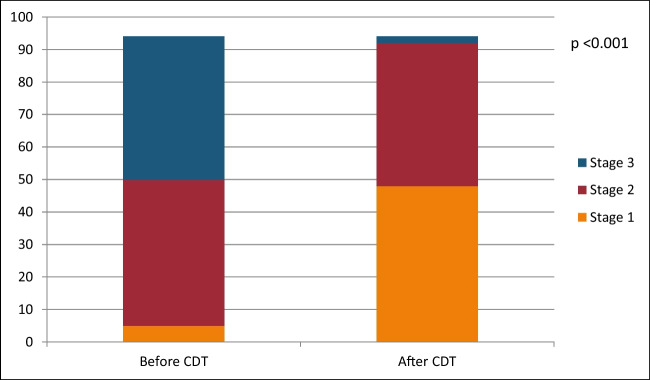
Lymphedema stages following CDT

As a result of CDT, posttreatment evaluation revealed that 48 patients were in stage 1, 34 patients were in stage 2, and 2 patients were in stage 3. These findings suggest that CDT had a positive impact on improving lymphedema stage in the majority of patients, with a significant number of patients experiencing an improvement in their lymphedema condition, particularly in stages 2 and 3 (Chart 1).

The correlation of HCFS score, PSQI Global score, LYMQOL-ARM score, and Global health status from baseline to the end of CDT treatment With Lymphedema Stage is shown in Table 5. The study found a statistically significant correlation between HCFS (Hirai Cancer Fatigue Scale) scores and Global health status scores with the lymphedema stage.

**Table 5:** Correlation of HCFS score, PSQI Global score, LYMQOL- ARM score, Global health status from baseline to the end of CDT treatment With Lymphedema Stage

	Lymphedema stages
HCFS score	*r* = 0.212, *p* = 0.040
PSQI Global Score	*r* = 0.112, *p* = 0.283
LYMQOL-ARM score	*r* = 0.144, *p* = 0.167
Global health status	*r* = − 0.246, *p* = 0.017

*Pearson correlation, *HCFS*, Hirai Cancer Fatigue Scale; *PSQI*, Pittsburgh Sleep Quality Index; *LYMQOL*, Lymphedema-specific Quality of Life; CDT, complete decongestive therapy

## Discussion

Our prospective study aimed to assess lymphedema-specific health-related quality of life, fatigue and sleep quality in breast cancer patients who developed secondary lymphedema after breast cancer surgery before and after CDT.

The results of the study indicate that postmastectomy lymphedema has a significant impact on global health status, with a decrease observed in all three stages of lymphedema. Specifically, the worst mean scores for LYMQOL-ARM and global health status were observed in patients with stage III lymphedema, indicating a higher symptom burden and reduced health-related quality of life in this group. Interestingly, the worst mean score for the PSQI Global, which measures sleep quality, was observed in patients with stage II lymphoedema. This suggests that sleep disturbance may be particularly common in this group, even though their lymphedema may not be as severe as in stage III patients.

These findings highlight the importance of addressing the impact of postmastectomy lymphedema on patients’ health-related quality of life, fatigue and sleep quality. The significant decrease in global health status across all lymphedema stages highlights the need for comprehensive management and support for breast cancer survivors who develop lymphedema. Furthermore, the emphasis on sleep disturbance in patients with stage II lymphedema may warrant additional attention to sleep interventions in this subgroup.

It would be beneficial to explore potential factors that contribute to differences in sleep quality among patients with different stages of lymphoedema. Understanding these factors may help to tailor interventions to more effectively address sleep disturbance in breast cancer survivors with lymphedema.

One study showed that participants’ health-related quality of life and sleep quality were influenced by several factors, including age, BMI, economic status, family status, and time since mastectomy [18]. This suggests that demographic and clinical factors may play an important role in the well-being of breast cancer survivors. In addition, another study showed that obesity was a risk factor for the development of lymphedema [19]. However, the same statistical significance was not found in our study.

This study also found that family status plays an important role in health-related quality of life and sleep quality. The impact of cultural factors, such as strong family ties and social support in Turkish culture, on the emotional and social well-being of cancer survivors during the treatment process may also contribute to the observed results.. In particular, social support from family members has been shown to positively affect the psychological well-being of cancer patients, including breast cancer survivors. Emotional and practical support from loved ones can help individuals cope with the challenges of cancer treatment, manage stress, and improve overall health-related quality of life.

In addition, the presence of a close-knit community and social support network can contribute to a sense of belonging and connectedness, which can have a positive impact on mental health and emotional well-being. It is essential to recognise the importance of cultural and social factors in understanding the experiences and outcomes of cancer survivors, as these factors may vary between regions and communities. In our study, 80 (85.1%) patients lived with their husbands, children or parents.

In our study, the significant changes observed in various outcome measures of CDT suggest that CDT may have positive effects on various aspects of participants’ health and well-being. This is consistent with previous research on Manual Lymphatic Drainage (MLD), a component of CDT, showing significant reductions in lymph volume and improvements in arm parameters and symptoms related to edema [20]. Likewise, in a study evaluating the efficacy and safety of CDT, patients experienced a significant reduction in breast cancer-related lymphedema with CDT alone [21].

For instance, Williams et al. conducted a randomised control crossover study in women and found that MLD significantly reduced limb volume and skin thickness. In additional, participants’ health-related quality of life and emotional function improved with this technique [22].

The significant changes observed in these outcome measures provide promising evidence for the effectiveness of CDT in improving the well-being of breast cancer survivors with lymphedema. These findings highlight the potential value of incorporating CDT into comprehensive cancer rehabilitation programmes to address the multiple challenges faced by breast cancer survivors and improve their overall health-related quality of life. However, further research with larger sample sizes and longer follow-up periods may be needed to validate and generalise these findings. A single-centre retrospective study analysed 81 patients with postmastectomy lymphedema and compared the results of lymphatic-venous anastomosis and CDT treatment. When comparing the rates of change in lymphedema, the therapeutic effect was significantly greater in the surgical treatment group than in the conservative treatment group, while lymphedema complications such as cellulitis were less common in the surgical group [23].

It’s promising that the majority of patients in our study have been downstaged in terms of the stage of the lymphedema. Downstaging refers to the improvement or regression of lymphedema stage, indicating that the condition is responding positively to the treatment or intervention. Numerous studies have shown that CDT is an effective intervention for reducing arm volume. As mentioned in previous research, CDT, including MLD and compression bandaging, is an effective intervention in reducing arm volume and improving symptoms associated with edema. The McNeely et al. findings that compression bandaging alone can be considered the primary treatment option for reducing lymphedema volume are consistent with the efficacy of CDT in the treatment of conditional lymphedema [24].

In a study, Ciudad et al. suggested that preoperative and postoperative CDT treatment is mandatory for all patients regardless of lymphedema stage, but additional excisional surgery (lymphatic-venous anastomosis, vascularised lymph node transfer, combined suction-assisted lipectomy) should be performed in advanced lymphedema patients [25]. Downstaging of lymphedema stages may indicate the effectiveness of CDT in managing and reducing lymphedema in breast cancer survivors. Seeing downstaging in a majority of patients suggests that the treatment is contributing to the reduction of lymphedema severity, leading to better outcomes for the patients.

Although many studies have been conducted to assess fatigue in patients previously treated for breast cancer, the effect of CDT on fatigue in postmastectomy lymphedema has not been evaluated [26–28]. The correlation between cancer-related fatigue and lymphedema stage indicates that as the severity of lymphedema increases, the level of fatigue experienced by patients tends to increase. This finding suggests that lymphedema can affect a patient’s overall energy and well-being. In a study of 202 breast cancer patients who had completed treatment, fatigue and health-related quality of life were assessed. Patients with fatigue were found to have a lower overall quality of life, which improved after CDT [29]. This study supports the literature and shows that patients with advanced stage lymphedema (ISL stage 3) have lower quality of life and higher levels of fatigue, suggesting that cancer-related fatigue can have a significant impact on the overall well-being and health-related quality of life of breast cancer survivors, highlighting the importance of addressing and managing fatigue in post-treatment care.

Lymphedema-related symptoms and discomfort can contribute to sleep disturbance in these patients. In patients with postmastectomy lymphedema syndrome, situations such as elevating the affected arm by placing a pillow underneath it, inability to change position during sleep, pain and swelling can negatively affect sleep quality [30]. These conditions may make it difficult for patients to find a comfortable sleeping position and may cause discomfort during sleep. It is important to use appropriate treatments and relaxation techniques to improve sleep quality [31]. The significant correlation between global health status and lymphedema stage suggests that as lymphedema severity increases, patients’ overall health and well-being tend to be negatively affected. This emphasises the need for comprehensive management and treatment of lymphedema to improve the patient’s overall health and quality of life. Although a recent study in a small group of patients did not find a significant improvement in sleep quality with CDT [32], a strong significant improvement in sleep quality was observed in our study. In the study by Gonzalez et al., sleep disturbance was more common in breast cancer patients with lymphedema, which supports our study [33].

Overall, these significant correlations highlight the interrelationship between cancer-related fatigue, sleep quality, lymphedema-specific quality of life, global health status and lymphedema stage severity in breast cancer survivors. Understanding these relationships can guide healthcare providers in developing targeted interventions to address and manage multiple aspects of lymphedema and its impact on the well-being of patients.

The strengths of our study can be defined as being the first study investigating the relationship between HCFS score, PSQI Global score, and CDT in postmastectomy patients with a large patient population and creating standardization by performing all measurements and treatments by the same people.

However, there are some limitations in this study. The cross-sectional design used in the study allows the assessment of several variables at a single point in time. However, this design cannot establish causality or determine the direction of relationships between variables. The sample size of 94 participants can be considered small for drawing generalisable conclusions. A larger sample size would increase the statistical power of the study and improve the representation of the whole population of women with breast cancer-related lymphedema. The study participants were recruited from a specific clinical setting, which may introduce selection bias. Including participants from different centres or regions would provide a more diverse and representative sample. The study relied solely on subjective assessment tools to collect data on cancer-related fatigue, sleep quality, lymphedema-specific quality of life and global health status. Objective assessment methods, such as physical measurements or biomarkers, could provide additional insights and complement the findings. The lack of a control group in the study makes it difficult to compare the observed results with a reference population. Including a control group of breast cancer survivors without lymphedema could help to better understand the specific impact of lymphedema on the variables of interest. There may be other unmeasured factors that could influence the relationship between the study variables. Controlling for potential confounders in the analysis would strengthen the results of the study.

Despite these limitations, the current study provides valuable insights into the relationship between cancer-related fatigue, sleep quality, lymphedema-specific quality of life, global health status and lymphedema stage in breast cancer survivors. Future research with larger sample sizes, longitudinal designs, and a combination of subjective and objective assessment tools would contribute to a more comprehensive understanding of the complex interactions and potential interventions for breast cancer-related lymphedema.

## Conclusions and recommendations

Cancer-related fatigue and sleep disturbance can persist for years after surgery in women with breast cancer. This can negatively affect the patient physically, socially and cognitively. Significant improvement was detected in the lymphedema stages of the patients included in the study after CDT. Before CDT, patients with advanced lymphedema had lower quality of life and higher fatigue levels. After CDT, patients’ sleep quality improved, fatigue levels decreased, general quality of life and global health status improved. Replicating and expanding the findings of this study is crucial to strengthen the evidence and gain a deeper understanding of the impact of breast cancer-related lymphedema on health-related quality of life and sleep. Additionally, identifying the risk factors that affect these outcomes in women with lymphedema can provide valuable insights for targeted interventions and improved patient care. Exploring the role of supportive care interventions, such as counseling, education, and psychosocial support, in mitigating the impact of lymphedema on health-related quality of life and sleep can be beneficial in enhancing the overall well-being of patients.

## Data Availability

No datasets were generated or analysed during the current study.
